# Thromboembolic and bleeding events after valvular intervention in patients with atrial fibrillation

**DOI:** 10.1136/openhrt-2024-002602

**Published:** 2024-01-29

**Authors:** Ebba-Louise Skogseid, Gorav Batra, Johan Westerbergh, Claes Held, Christina Christersson

**Affiliations:** 1 Department of Medical Sciences, Cardiology, Uppsala University, Uppsala, Sweden; 2 Uppsala Clinical Research Center, Uppsala University, Uppsala, Sweden

**Keywords:** Heart Valve Diseases, Heart Valve Prosthesis, Atrial Fibrillation, Transcatheter Aortic Valve Replacement, Heart Valve Prosthesis Implantation

## Abstract

**Aim:**

To assess outcomes after cardiac surgery with biological valve replacement, valve repair or transcatheter aortic valve implantation (TAVI) in patients with atrial fibrillation (AF) in accordance with oral anticoagulant (OAC) treatment.

**Methods:**

All patients in Sweden undergoing valvular intervention with AF were included. Associations between OAC exposure and cardiovascular (CV) events (composite of CV death, ischaemic stroke or systemic embolism) and major bleeding were investigated using Cox regression analysis. The analysis was separated in time periods of 0–3 and 3–12 months after discharge.

**Results:**

4730 patients were included in the first time period, 54.0% had received a surgical biological valve prosthesis, 23.8% valve repair and 22.2% TAVI. Exposure to warfarin (comparator) was 62.3%, to non-vitamin K antagonist oral anticoagulants (NOACs) 10.0% and to no OAC 27.7%. NOAC exposure was associated with similar risk of the composite CV outcome and major bleeding from 0 to 3 months. No OAC was associated with increased risk of the composite CV outcome (HR 1.71; 95% CI 1.26 to 2.32) and similar risk of major bleeding. Further analysis of the bioprosthetic valve replacement subgroup indicated increased risk of CV death when exposed to NOAC (HR 2.58; 95% CI 1.15 to 5.78) and no OAC (HR 2.82; 95% CI 1.65 to 4.82) compared with warfarin from 0 to 3 months. No differences were seen between 3 and 12 months.

**Conclusion:**

In this registry-based cohort study of patients with AF with severe valvular heart disease undergoing various valvular interventions, NOAC appears to be comparable with warfarin regarding efficacy and safety. Patients not receiving OAC had higher risk of CV events. NOAC was associated with increased CV death compared with warfarin in the surgical bioprosthetic valve replacement subgroup, illustrating the importance of being cautious when extrapolating data from one patient group to another. Further studies comparing NOAC and warfarin in the early postoperative phase are warranted, especially following surgical bioprosthetic valve replacement.

**WHAT IS ALREADY KNOWN ON THIS TOPIC**

- Randomised controlled trials comparing non-vitamin K antagonist oral anticoagulants (NOACs) with warfarin in patients undergoing biological valve intervention with coexisting atrial fibrillation (AF) are sparse. The number of patients included in the first 90 days following intervention is limited.

**WHAT THIS STUDY ADDS**

- NOAC appears to be comparable with warfarin with similar efficacy and safety in a mixed cohort of patients with AF with various types of severe valvular heart disease during the first year following valvular intervention. NOAC was associated with increased risk of cardiovascular death in patients undergoing surgical valve replacement with biological prosthesis compared with warfarin during the first 90 days following intervention.

**HOW THIS STUDY MIGHT AFFECT RESEARCH, PRACTICE OR POLICY**

- This study highlights the importance of adherence to clinical guidelines for patients with AF and that further prospective studies comparing NOAC and warfarin in patients with AF early after valvular intervention are warranted, especially following surgical biological valve replacement.

## Introduction

 Patients with atrial fibrillation (AF) and valvular heart disease (VHD) have increased risk of stroke, systemic embolism and mortality compared with patients with AF without VHD.^1^,^5^ A biological valve prosthesis is less thrombogenic than a mechanical one, but there remains an increased risk of thromboembolic events, particularly in the early postoperative phase and especially among patients with coexisting AF.^6^,^8^ There has been a controversy as to whether non-vitamin K antagonist oral anticoagulants (NOACs) and warfarin are comparable for primary prevention of thromboembolism in patients with AF and a coexisting biological valve prosthesis.^9 10^ Few randomised controlled trials (RCTs) specifically designed to compare NOAC with warfarin in patients with AF and biological valve prosthesis have been conducted. Available trial results indicate non-inferiority with regard to major cardiovascular (CV) events and bleeding, except for edoxaban which was associated with increased bleeding.^11^,^14^

The aim of this nationwide observational cohort study was to describe the risk of CV and major bleeding events during the first year after cardiac surgery with biological valve replacement, valve repair or transcatheter aortic valve implantation (TAVI) in patients with coexisting AF in accordance with oral anticoagulant (OAC) treatment.

## Methods

### Study population

The study cohort was identified in the Swedish Web-system for Enhancement and Development of Evidence-based care in Heart disease Evaluated According to Recommended Therapies (SWEDEHEART) registry. SWEDEHEART is a national quality registry containing individualised data on all patients undergoing valvular intervention in Sweden. All patients, 18 years or above, undergoing surgical biological valve replacement, valve repair or TAVI in Sweden between 1 January 2010 and 31 December 2016 with coexisting AF previously known at the time of discharge from index valve intervention or else diagnosed within the first 3 postoperative months, were eligible for the study. Patients with mechanical heart valves or who died before discharge were excluded.

### Data collection

Baseline data were obtained from SWEDEHEART, supplemented with information from the National Patient Register (NPR), which contains diagnosis codes from all hospital admissions in Sweden based on the International Classification of Diseases, 10th revision. Outcome events occurring during follow-up were collected using NPR and the National Cause of Death Register, whereas information about drug prescriptions and dispensations was procured from the National Prescribed Drug Register. Linkage was based on the unique 10-digit personal identification number assigned to all Swedish residents at birth or immigration, all managed by the National Board of Health and Welfare in Sweden. Patients were monitored via their unique personal identifier through the above-mentioned registries from inclusion until death or end of follow-up.

### Patient characteristics and comorbidities

Comorbidities were continuously monitored and updated during follow-up (online supplemental table 1). The CHA_2_DS_2_-VASc (online supplemental table 2) and HAS-BLED scores (online supplemental table 3) were calculated for each study participant based on their clinical characteristics and medical history.

### Exposure to OAC

When listing clinical characteristics at baseline, a patient was regarded as exposed to OAC if: (1) OAC was dispensed either within 7 days from discharge or (2) if OAC had been dispensed prior to intervention and the prescription was renewed post-discharge within 120 days. For the regression analysis, OAC exposure was defined through on-treatment analysis where a patient was regarded as exposed to an OAC for 120 days following drug dispensation and thereafter considered to be without treatment unless the prescription was renewed. If a new type of OAC was dispensed later, the patient changed treatment group in the analysis. Consequently, each patient could potentially be exposed to several different OAC medications during different time points in the follow-up period. The different exposures in this study were (1) warfarin, (2) NOAC or (3) no OAC (online supplemental table 4).

### Outcome events

The primary outcome was a composite of CV death, ischaemic stroke or systemic embolism. Secondary outcomes included the composite of ischaemic stroke and systemic embolism, and the individual outcome of CV death. The main safety outcome was major bleeding (online supplemental table 5).

### Statistical methods

Throughout the study, two separate study cohorts were defined, groups A and B. In group A, patients with previously known AF at the time of discharge from index valve intervention were included. Analysis of outcome events occurring during the first 3 months following valve intervention was based on group A. Perioperative events were not included in the analysis. Group B consisted both of patients with previously known AF and those diagnosed with AF during the first 3 months of follow-up. Group B was used when analysing outcome events taking place from 3 months up to 1 year after index valve intervention. Descriptive statistics were calculated for the two separate cohorts—groups A and B. Categorical variables were described as frequencies and percentages and the continuous variables expressed as medians with IQRs. The study populations’ exposure to each treatment group was expressed in patient years (PY). Outcome events were expressed as incidence rate per 100 PY (%/year) and graphically illustrated as Simon-Makuch modified Kaplan-Meier plots in an on-treatment analysis.^15^ The association between exposure (warfarin, NOAC, no OAC) and occurrence of outcome events was explored using a time-dependent Cox regression analysis. The NOAC and no OAC exposure groups were compared against those exposed to warfarin. Two models were implemented: a crude model for each outcome event in which OAC exposure was the only time-dependent variable, and an adjusted model including baseline characteristics (age, sex, estimated glomerular filtration rate based on the Modification of Diet in Renal Disease formula and plasma creatinine) and comorbidities (hypertension, diabetes mellitus, peripheral arterial disease, heart failure, previous myocardial infarction, ischaemic stroke, transient ischaemic attack, unspecified stroke, systemic embolism, venous thromboembolism and major bleeding), continuously updated (online supplemental table 1). For the outcome major bleeding, adjustments were also made for ongoing antiplatelet treatment. The results of the crude and adjusted Cox regression analysis were presented as HRs with 95% CI. In addition, we also repeated the above-mentioned time-dependent Cox regression analysis dichotomising exposure to OAC or no OAC.

We also performed subgroup analyses of the patients undergoing surgical valve intervention with a biological valve prosthesis or TAVI, respectively. Analysis of interaction effect of OAC exposure and valve position (aortic or mitral) on risk of thromboembolic and bleeding events was performed in the bioprosthesis subgroup. All tests were two sided and p value of <0.05 implied statistical significance. Statistical analyses were performed using R statistics V.3.5.0 (The R Foundation for Statistical Computing).

### Patient and public involvement

The patients were not involved in the present study.

## Results

### Study cohorts and baseline characteristics

**Figure 1:**
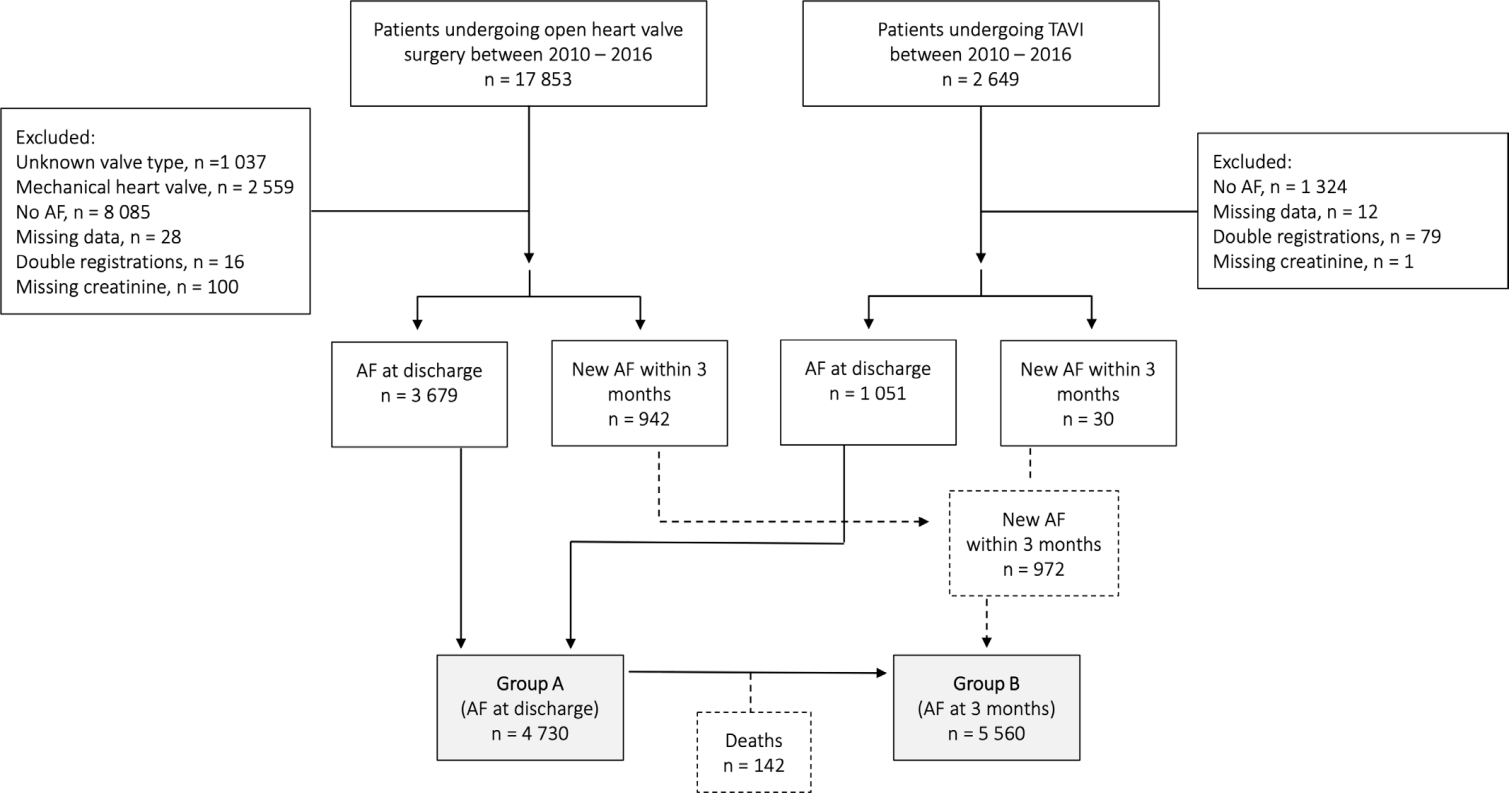
Description of the study cohorts. AF, atrial fibrillation; TAVI, transcatheter aortic valve implantation.

The study cohort was identified in SWEDEHEART and included all patients who underwent surgical valvular intervention or TAVI between the years 2010 and 2016 with AF (figure 1). Group A consisted of 4730 patients and group B 5560. Baseline characteristics of group A are described in table 1. The proportion of patients with chronic kidney failure was slightly higher in patients without OAC treatment compared with warfarin or NOAC, and the prevalence of previous myocardial infarction, stroke or gastrointestinal bleeding was marginally higher in the no OAC and NOAC group compared with warfarin. For baseline data of group B, see online supplemental table 6. The proportion of different NOACs is described in table 2.

**Table 1:** Baseline characteristics of group A

	All patients	Warfarin	NOAC	No OAC
	N=4730	N=2068	N=348	N=2314
Age, median (IQR)	75 (68–81)	74 (67–79)	76 (70–82)	76 (69–81)
Female sex, median (IQR)	1669 (35.9)	665 (32.2)	128 (36.8)	906 (39.2)
Creatinine (µmol/L), median (IQR)	101 (81–134)	97 (79–125)	98 (81–120)	105 (84–130)
**Medical history**				
Heart failure, no (%)	1797 (38.0)	740 (35.8)	131 (37.6)	926 (40.0)
Hypertension, no (%)	2532 (53.5)	1064 (51.5)	185 (53.2)	1283 (55.4)
Diabetes mellitus, no (%)	797 (16.8)	324 (15.7)	48 (13.8)	425 (18.4)
Ischaemic stroke, no (%)	323 (6.8)	122 (5.9)	28 (8.0)	173 (7.5)
TIA, no (%)	204 (4.3)	84 (4.1)	14 (4.0)	106 (4.6)
Myocardial infarction, no (%)	739 (15.6)	285 (13.8)	58 (16.7)	396 (17.1)
Peripheral artery disease, no (%)	349 (7.4)	133 (6.4)	25 (7.2)	191 (8.3)
Systemic embolism, no (%)	33 (0.7)	20 (1.0)	0 (0.0)	13 (0.6)
Chronic kidney disease, no (%)	322 (6.8)	94 (4.5)	20 (5.7)	208 (9.0)
Cancer, no (%)	239 (5.1)	90 (4.4)	15 (4.3)	134 (5.8)
Mitral stenosis, no (%)	85 (1.8)	36 (1.7)	2 (0.6)	47 (2.0)
Intracranial bleeding, no (%)	60 (1.3)	19 (0.9)	6 (1.7)	35 (1.5)
Gastrointestinal bleeding, no (%)	299 (6.3)	93 (4.5)	27 (7.8)	179 (7.7)
Other major bleeding, no (%)	333 (7.0)	111 (5.4)	20 (5.7)	202 (8.7)
CHA_2_DS_2_-VASc score, mean (SD)	3.3 (1.8)	3.1 (1.8)	3.4 (1.6)	3.5 (1.8)
HAS-BLED, mean (SD)	1.7 (0.9)	1.6 (0.9)	1.7 (0.8)	1.7 (0.9)
**Valve intervention type**				
Surgical biological valve prosthesis, no (%)	2554 (54.0)	1078 (52.1)	164 (47.1)	1312 (56.7)
Surgical valvuloplasty, no (%)	1125 (23.8)	621 (30.0)	56 (16.1)	448 (19.4)
TAVI, no (%)	1051 (22.2)	369 (17.8)	128 (36.8)	554 (23.9)
**Valve position**				
Aortic position, no (%)	3389 (71.6)	1345 (65.0)	290 (83.3)	1754 (75.8)
Mitral position, no (%)	1374 (29.0)	749 (36.2)	61 (17.5)	564 (24.4)
Tricuspid position, no (%)	584 (12.3)	262 (12.7)	20 (5.7)	302 (13.1)
Pulmonary position, no (%)	23 (0.5)	4 (0.2)	0 (0.0)	19 (0.8)

NOAC, non-vitamin K antagonist oral anticoagulant; OAC, oral anticoagulant; SD, standard deviation; TAVI, transcatheter aortic valve implantation; TIA, transient ischaemic attack.

**Table 2:** The proportion of different NOACs

	Previously known AF at time of discharge from valve intervention (group A)	Previously known or new-onset AF diagnosed during the first 3 months following valve intervention (group B)
NOAC	N=348	N=642
Apixaban	238 (68.4)	432 (67.3)
Edoxaban	0 (0.0)	0 (0.0)
Rivaroxaban	61 (17.5)	115 (17.9)
Dabigatran	49 (14.1)	95 (10.1)

AF, atrial fibrillation; NOACs, non-vitamin K antagonist oral anticoagulants.

### OAC exposure during follow-up

The proportion of exposure to warfarin in relation to person-time was 62.3% and to NOAC 10.0% during the first 3 months following valve intervention. Patients were without OAC 27.7% of the total person-time during the first 3 months. During the time period 3–12 months after valve intervention, the proportion of exposure to warfarin was 52.5%, to NOAC 13.4% and no OAC 34.1%.

### Primary outcome

**Figure 2:**
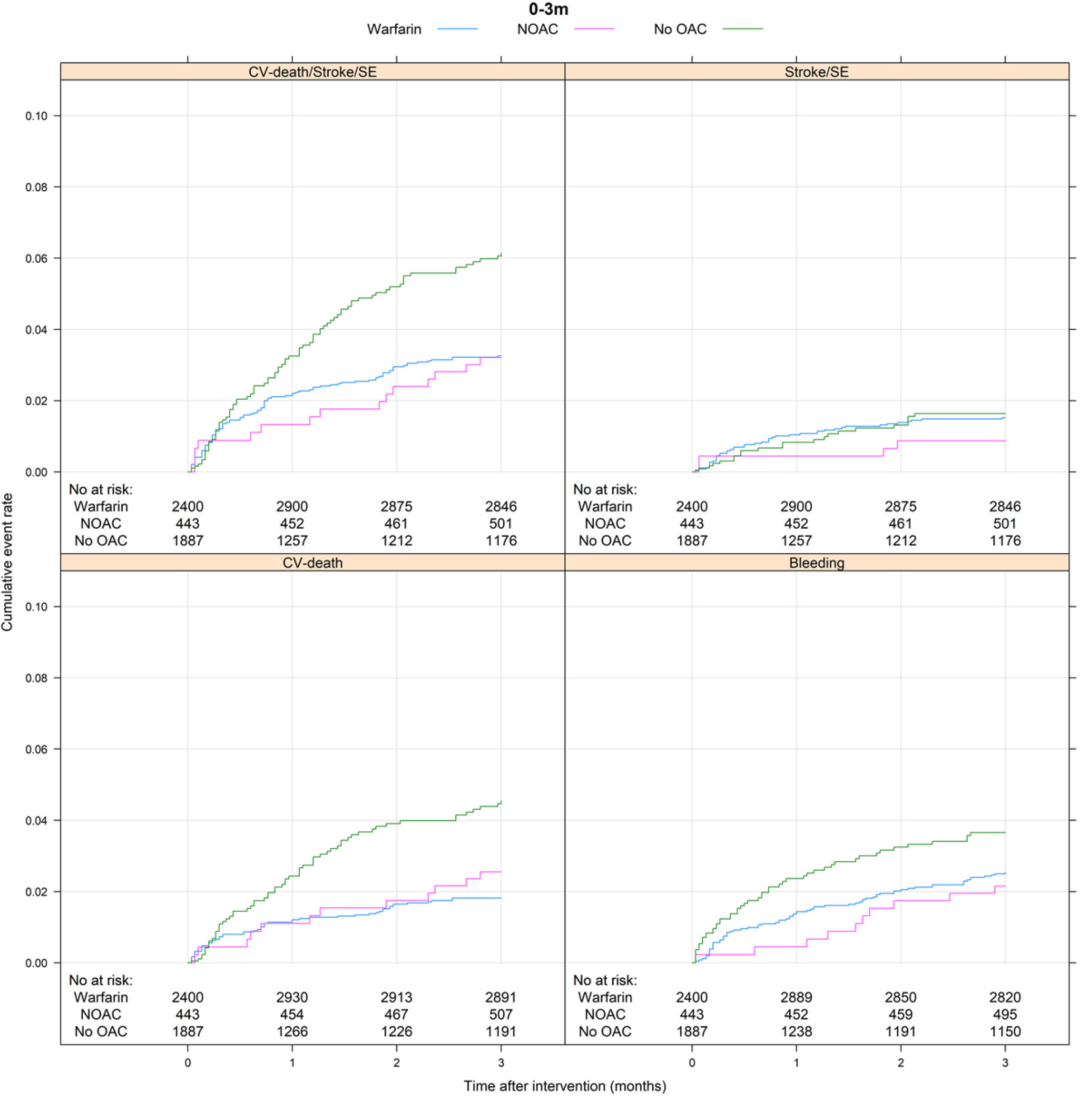
Cumulative event rate for the composite CV outcome (CV death, ischaemic stroke or systemic embolism (SE)); ischaemic stroke or SE; CV death and major bleeding during the first 3 months after discharge from valvular intervention for patients treated with warfarin, NOAC or no OAC. CV death, cardiovascular death; NOAC, non-vitamin K antagonist oral anticoagulant; No at risk, number at risk; OAC, oral anticoagulant; SE, systemic embolism.

**Figure 3:**
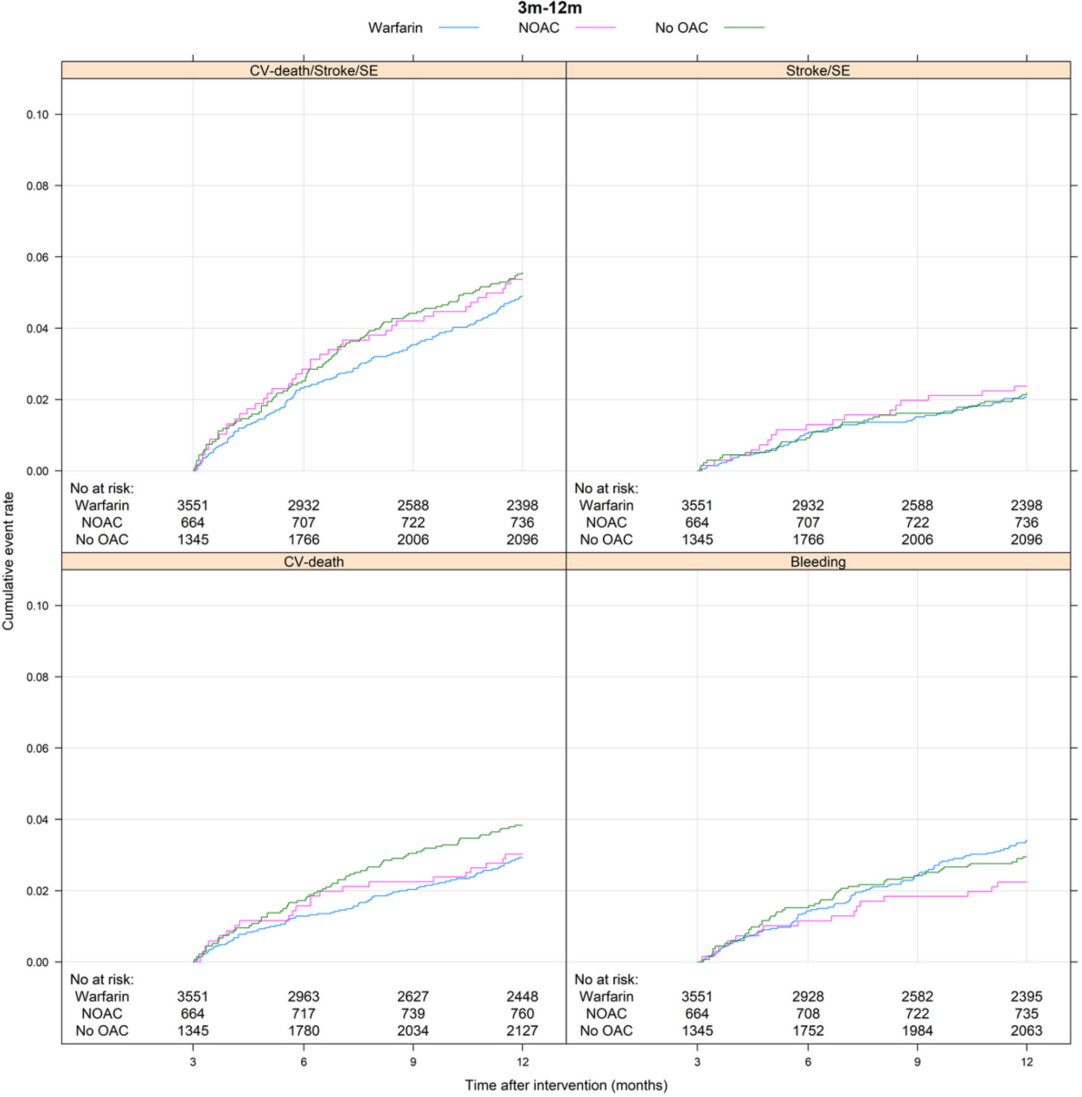
Cumulative event rate for the composite CV outcome (CV death, ischaemic stroke or systemic embolism (SE)); ischaemic stroke or SE; CV death and major bleeding from 3 to 12 months after valvular intervention separated by warfarin, NOAC or no OAC exposure. CV death, cardiovascular death; NOAC, non-vitamin K antagonist oral anticoagulant; No at risk, number at risk; OAC, oral anticoagulant; SE, systemic embolism.

**Figure 4:**
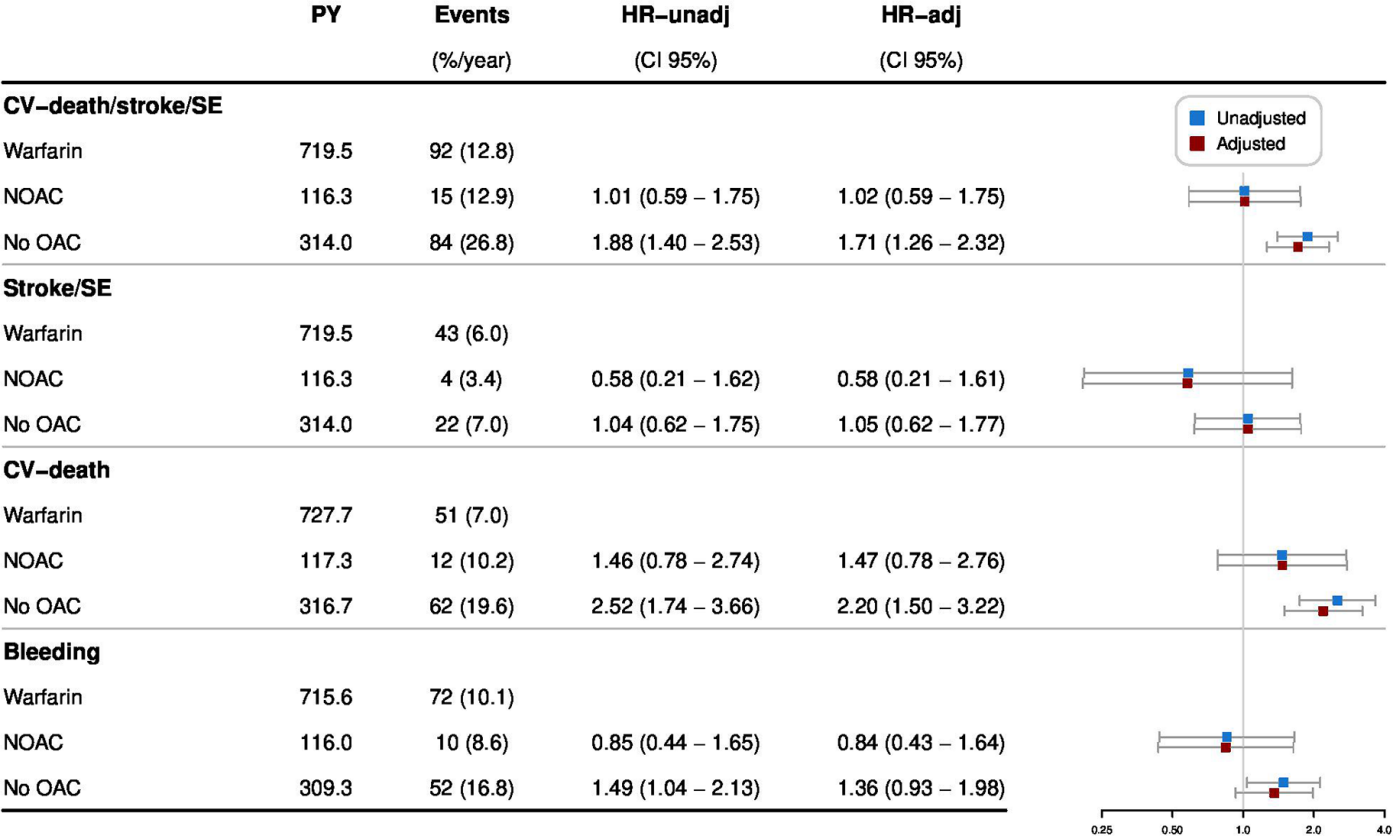
The event rates of the composite CV outcome (CV death, ischaemic stroke or systemic embolism (SE)); ischaemic stroke or SE; CV death and major bleeding, and HR (95% CI) for NOAC versus warfarin and no OAC versus warfarin during the first 3 months after discharge from valvular intervention. The adjusted model included baseline characteristics and comorbidities. %/year, per cent per PY; CV death, cardiovascular death; HR-adj, HR adjusted; HR-unadj, HR unadjusted; NOAC, non-vitamin K antagonist oral anticoagulant; OAC, oral anticoagulant; PY, patient years; SE, systemic embolism.

**Figure 5:**
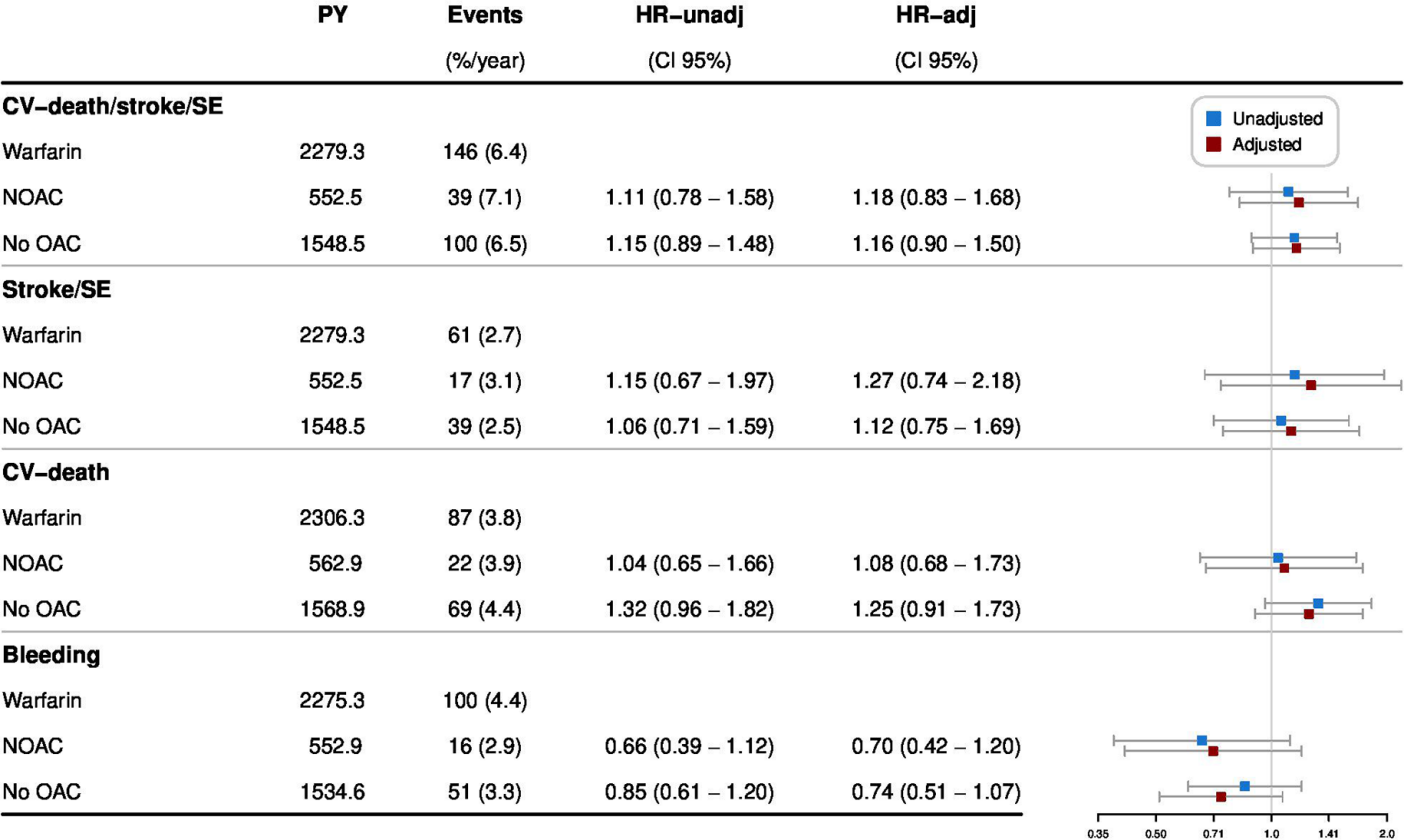
The event rates of the composite CV outcome (CV death, ischaemic stroke or systemic embolism (SE)); ischaemic stroke or SE; CV death and major bleeding, and HR (95% CI) for NOAC versus warfarin and no OAC versus warfarin from 3 to 12 months after the valvular intervention. The adjusted model included baseline characteristics and comorbidities. %/year, per cent per PY; CV death, cardiovascular death; HR-adj, HR adjusted; HR-unadj, HR unadjusted; NOAC, non-vitamin K antagonist oral anticoagulant; OAC, oral anticoagulant; PY, patient years; SE, systemic embolism.

During the first 3 months following intervention, the composite CV outcome occurred in 191 patients, of which 92 in patients exposed to warfarin (12.8%/year), 15 in patients exposed to NOAC (12.9%/year) and 84 in patients with no OAC treatment (26.8%/year) (table 3 and figure 2). In comparison, 285 events occurred at 3–12 months following intervention, 146 when on warfarin (6.4%/year), 39 when on NOAC (7.1%/year) and 100 in patients without OAC (6.5%/year) (table 3 and figure 3). For the Cox regression analysis, we saw similar risk of the primary outcome occurring while exposed to NOAC and higher risk in patients without OAC treatment (HR 1.71; 95% CI 1.26 to 2.32) as compared with patients on warfarin in the adjusted model during the first 3 months after intervention (figure 4). No differences were found between 3 and 12 months (figure 5). For results from Cox regression analysis dichotomising exposure to no OAC versus OAC, see online supplemental figures 1 and 2.

**Table 3:** Oral anticoagulant exposure and outcome during follow-up

Time period	Discharge to 3 months post-intervention			3–12 months post-intervention		
	Warfarin	NOAC	No OAC	Warfarin	NOAC	No OAC
[Table-fn T3_FN1]CompositeCV event*	92 (719) 12.8	15 (116) 12.9	84 (314) 26.8	146 (2279) 6.4	39 (553) 7.1	100 (1549) 6.5
[Table-fn T3_FN1]CV death*	51 (728) 7.0	12 (117) 10.2	62 (317) 19.6	87 (2306) 3.8	22 (563) 3.9	69 (1568) 4.4
Ischaemic stroke/SE*	43 (719) 6.0	4 (116) 3.4	22 (314) 7.0	61 (2279) 2.7	17 (553) 3.1	29 (1549) 2.5
[Table-fn T3_FN1][Table-fn T3_FN1]Major bleeding*	72 (716) 10.1	10 (116) 8.6	52 (309) 16.8	100 (2275) 4.4	16 (553) 2.9	51 (1535) 3.3

* Expressed as frequencies (patient years), incidence rate, %/year.

CV, cardiovascular; NOAC, non-vitamin K antagonist oral anticoagulant; SE, systemic embolism.

### Secondary outcomes

The secondary outcomes ischaemic stroke or systemic embolism and CV death separated by OAC exposure are described in table 3 and figures2^5^.

### OAC exposure and major bleeding

During the first 3 months after valve intervention, 134 patients had a major bleeding, of which 72 while exposed to warfarin (10.1%/year), 10 to NOAC (8.6%/year) and in 52 with no OAC treatment (16.8%/year) (table 3 and figure 2). Between 3 and 12 months, 167 patients had major bleeding: 100 on warfarin (4.4%/year), 16 on NOAC (2.9%/year) and in 51 patients without OAC (3.3%/year) (table 3 and figure 3). There were no differences in risk of major bleeding during any time point when comparing the different OAC exposure groups in the adjusted model (figures4  5).

### Subgroup analyses

Patients who underwent surgical valve replacement with biological prostheses had similar risk of the primary outcome occurring on NOAC and higher risk without OAC (HR 1.88; 95% CI 1.25 to 2.84) as compared with warfarin the first 3 months following valve intervention in the adjusted model (online supplemental figure 3 and table 7). No difference was seen in risk of major bleeding. There was increased risk of the secondary outcome CV death during the first 3 months in both NOAC (2.58; 95% CI 1.15 to 5.78) and no OAC (HR 2.82; 95% CI 1.65 to 4.82) compared with warfarin. No difference was seen in any outcome from 3 to 12 months after valve intervention (online supplemental figure 4 and table 7). No interaction effect of OAC treatment and valvular position (mitral and aortic) on the risk of thromboembolic or bleeding events was identified (online supplemental table 8).

When analysing TAVI patients separately, there was increased risk of bleeding in patients without OAC compared with warfarin in the adjusted model in the early postoperative phase (online supplemental figure 5). No other differences were seen in any outcome at either time point (online supplemental table 9 and figures 5 and 6).

## Discussion

This nationwide observational cohort study evaluated patients with AF with various types of severe VHD undergoing valve intervention. The incidence of thromboembolic and major bleeding events was higher during the first 3 months after discharge from valvular intervention compared with the time period between 3 and 12 months post-intervention. Perioperative events were not included in the analysis. For our main analysis, we included patients with AF undergoing surgical valvular replacement, valve repair and TAVI. Our results from this combined group indicate that NOAC is comparable with warfarin both regarding the risk of CV and major bleeding events during the early postoperative phase, as well as from 3 months up to 1 year following valve intervention. Compared with warfarin, no OAC treatment was associated with a higher risk of CV events, including CV death early after valve intervention. However, when analysing the subgroup of surgical valve replacement separately, both NOAC and no OAC were associated with significantly increased risk of CV death in the early postoperative phase compared with warfarin. In TAVI patients, no OAC treatment was associated with significantly increased risk of bleeding compared with warfarin during the first 3 months following intervention.

At present, few large prospective RCTs have been conducted in patients with AF undergoing biological valve intervention. Among available trial results, the Rivaroxaban for Valvular Heart Disease and Atrial Fibrillation (RIVER) trial included patients with AF and biological mitral valve prosthesis randomised to either rivaroxaban or warfarin. Rivaroxaban was non-inferior to warfarin with respect to the risk of CV and major bleeding events.^11^ Among the subgroup of patients receiving rivaroxaban randomised during the early postoperative period, the incidence rate of the outcome events was lower compared with warfarin. However, the small number of patients in this subgroup casts uncertainty to this finding. The open randomised controlled study Efficacy and safety of Edoxaban in patients early after surgical bioprosthetic valve implantation or valve repair (ENAVLE) indicated non-inferiority for edoxaban also in the subanalysis of patients with concomitant AF.^14^ A total of 218 patients were included in the study and the event rate was very low making it hard to reach any conclusions based on the results. In the present study, the subanalysis of patients with AF with surgical bioprosthetic valves showed an association between CV death and exposure to NOAC as well as no OAC compared with patients exposed to warfarin during the first 3 postoperative months. No conclusions can be drawn based on these results but they emphasise the gap of evidence in treatment recommendations in patients with AF early after surgical valve intervention and the importance of improving our knowledge of efficacy and safety of NOAC in patients with AF upon intervention with surgical biological valve prosthesis.^9 10 16^

The RCTs comparing factor Xa inhibitors with warfarin after TAVI, in patients with other indication for OAC, have not shown any advantage of NOAC compared with warfarin.^12 13^ The results even indicated that edoxaban was associated with an increased risk of major bleeding compared with warfarin, which was not found when comparing apixaban with warfarin.^12 13^ In the present study, the bleeding risk associated with NOAC was similar to warfarin both in the early and late phase during the first year after valvular intervention. The main advantage of NOACs in the phase III RCTs in patients with non-valvular AF was the lower risk of major bleeding compared with warfarin, results which have yet not been confirmed in patients with recent valvular intervention.^17 18^ However, patients undergoing valvular intervention are often older, with higher proportions of comorbidities and represent a more fragile group compared with patients with non-valvular AF, which might contribute to the higher occurrence of bleeding with NOAC treatment.^19 20^ Some studies describe differences in major bleeding when comparing various NOACs in elderly patients with non-valvular AF.^19 20^ Apixaban has been suggested to be related to lower risk of severe bleeding compared with rivaroxaban and dabigatran in real-world studies of patients with AF.^21 22^ The majority of the patients in the present study were treated with apixaban and whether different NOACs exert different risks in patients with AF undergoing valvular intervention needs to be further explored.

In the present study, the risk of thromboembolic events was higher in the early postoperative period after valve intervention compared with later during the first year. Both the increased inflammation and coagulation activity found in patients with valvular disease early after intervention might contribute to the benefit observed with OAC treatment in this study.^23 24^

A large proportion of patients in the current study did not receive OAC treatment despite guidelines clearly stating that patients with AF receiving bioprosthetic heart valves, valve repair or TAVI have an indication for OAC treatment.^9 10^ CHA_2_DS_2_-VASc is associated with increased risk of thromboembolic events in AF irrespective of biological valve prosthesis.^25^ In the current study, no differences could be observed between the treatment groups regarding estimated risk of stroke and bleeding according to the CHA_2_DS_2_-VASc and HAS-BLED scores at the time of intervention. Even if prescription of OAC in patients with AF has increased during the last decade, observational studies still show that improved adherence to guidelines for prescription of OAC can further reduce the risk of worse outcome.^26 27^ However, patients not exposed to OAC might be vulnerable, with both high thromboembolic and bleeding risk, and the uncertainty of how to best choose OAC after valvular intervention may contribute to physicians withholding OAC therapy at discharge. A previous study found that patients with AF left without OAC treatment following valvular intervention more often have chronic kidney failure, previous major bleeding and were of female sex compared with patients treated with OAC.^28^ Large prospective RCTs of OAC treatments focusing on patients with AF in the first 3 months after valve intervention are warranted to improve the knowledge of best treatment of these patients with high thromboembolic as well as bleeding risk.

The results in the TAVI subgroup differ from the results from the main analysis of the mixed intervention cohort as well as the surgical bioprosthetic valve replacement group. It is unclear whether this is a reflection of differences in perioperative procedure between TAVI and open-heart valve replacement and the unique structure of the TAVI prosthesis itself or rather a consequence of the TAVI patients being older with more comorbidities.

The present study has several limitations. Results are based on data from national registries and the identification of the study cohort in SWEDEHEART registry may imply some limitations about data quality. However, the SWEDEHEART registry has 100% complete coverage of unselected enrolment of patients undergoing surgical valve interventions and TAVI. The registry is subject to yearly random on-site monitoring and validation and has been shown to produce high levels of agreement between the registry and patient health records.^29^ The study does not differentiate between previously known and new-onset AF diagnosed before discharge from valve intervention. Systematic reviews and meta-analyses have identified increased risk of mortality and thromboembolic events in patients diagnosed with new-onset AF after cardiac surgery.^30^ However, defining new-onset AF is troublesome and previous studies have shown that a significant portion of patients admitted for cardiac surgery actually present with AF on preoperative ECG without it being previously known.^31^

In the current study, information from medical records on the reasoning behind choice of OAC strategy was not accessible due to the fact that the study is based solely on data from national quality registries. The measurements of the international normalised ratio of the patients prescribed warfarin were not possible to obtain from the data extraction from the registries. However, anticoagulation treatment in Sweden is generally of good quality with high time in therapeutic range.^32^ Drug exposure was defined through OAC dispensations. However, actual OAC intake could not be guaranteed.^33^ Pharmaceuticals are included in a comprehensive reimbursement programme in Sweden, and usage of pharmaceuticals outside this programme can be expected to be marginal.

Despite the limitations, our approach, which included a continuous update of pharmaceutical exposure during all follow-ups, should offer an accurate assessment of outcome associated with exposure to AF and valve intervention and related medication. Finally, our study has to be interpreted with caution because of inherent limitations to all observational studies related to possible confounding by indication.

In conclusion, our results indicate that NOAC appears to be a comparable alternative to warfarin with similar efficacy and safety following valve replacement with biological valve prosthesis, valve repair or TAVI in patients with AF. Patients not receiving OAC seem to have a higher risk of CV events, indicating the importance of adherence to guideline recommendations. Additional RCTs are warranted comparing NOAC and warfarin treatment early after valvular intervention and especially after surgical bioprosthetic valve replacement.

## Supplementary material

10.1136/openhrt-2024-002602 online supplemental file 1

## Data Availability

Data are available upon reasonable request.

## References

[1] Avezum A, Lopes RD, Schulte PJ (2015). Apixaban in comparison with warfarin in patients with atrial fibrillation and valvular heart disease: findings from the Apixaban for reduction in stroke and other thromboembolic events in atrial fibrillation (ARISTOTLE). *Circulation*.

[2] Halperin JL, Hart RG (1988). Atrial fibrillation and stroke: new ideas, persisting dilemmas. Stroke.

[3] Nabauer M, Gerth A, Limbourg T (2009). The Registry of the German competence network on atrial fibrillation: patient characteristics and initial management. Europace.

[4] Nieuwlaat R, Capucci A, Camm AJ (2005). Atrial fibrillation management: a prospective survey in ESC member countries: the Euro heart survey on atrial fibrillation. Eur Heart J.

[5] Philippart R, Brunet-Bernard A, Clementy N (2015). “Prognostic value of Cha2Ds2-Vasc score in patients with 'non-valvular atrial fibrillation' and valvular heart disease: the Loire valley atrial fibrillation project”. Eur Heart J.

[6] Heras M, Chesebro JH, Fuster V (1995). High risk of Thromboemboli early after Bioprosthetic cardiac valve replacement. J Am Coll Cardiol.

[7] Brennan JM, Edwards FH, Zhao Y (2012). Early anticoagulation of Bioprosthetic aortic valves in older patients: results from the society of Thoracic Surgeons adult cardiac surgery national database. J Am Coll Cardiol.

[8] Dangas GD, Weitz JI, Giustino G (2016). Prosthetic heart valve thrombosis. J Am Coll Cardiol.

[9] Hindricks G, Potpara T, Dagres N (2021). ESC guidelines for the diagnosis and management of atrial fibrillation developed in collaboration with the European Association for Cardio-Thoracic surgery (EACTS). Eur Heart J.

[10] Vahanian A, Beyersdorf F, Praz F (2022). ESC/EACTS guidelines for the management of valvular heart disease. Eur Heart J.

[11] Guimarães HP, Lopes RD, de Barros E Silva PGM (2020). Rivaroxaban in patients with atrial fibrillation and a Bioprosthetic mitral valve. N Engl J Med.

[12] Van Mieghem NM, Unverdorben M, Hengstenberg C (2021). Edoxaban versus vitamin K antagonist for atrial fibrillation after TAVR. N Engl J Med.

[13] Collet JP, Van Belle E, Thiele H (2022). Apixaban vs. standard of care after Transcatheter aortic valve implantation: the ATLANTIS trial. Eur Heart J.

[14] Shim CY, Seo J, Kim YJ (2023). Efficacy and safety of Edoxaban in patients early after surgical Bioprosthetic valve implantation or valve repair: a randomized clinical trial. J Thorac Cardiovasc Surg.

[15] Simon R, Makuch RW (1984). A non-parametric graphical representation of the relationship between survival and the occurrence of an event: application to Responder versus non-Responder bias. Stat Med.

[16] Steffel J, Collins R, Antz M (2021). European heart rhythm Association practical guide on the use of non-vitamin K antagonist oral anticoagulants in patients with atrial fibrillation. Europace.

[17] Carnicelli AP, De Caterina R, Halperin JL (2017). Edoxaban for the prevention of thromboembolism in patients with atrial fibrillation and Bioprosthetic valves. Circulation.

[18] Guimarães PO, Pokorney SD, Lopes RD (2019). Efficacy and safety of Apixaban vs warfarin in patients with atrial fibrillation and prior Bioprosthetic valve replacement or valve repair: insights from the ARISTOTLE trial. Clin Cardiol.

[19] Rutherford O-CW, Jonasson C, Ghanima W (2022). Effectiveness and safety of oral anticoagulants in elderly patients with atrial fibrillation. Heart.

[20] Silverio A, Di Maio M, Prota C (2021). Safety and efficacy of non-vitamin K antagonist oral anticoagulants in elderly patients with atrial fibrillation: systematic review and meta-analysis of 22 studies and 440 281 patients. Eur Heart J Cardiovasc Pharmacother.

[21] Halvorsen S, Ghanima W, Fride Tvete I (2017). A nationwide Registry study to compare bleeding rates in patients with atrial fibrillation being prescribed oral anticoagulants. Eur Heart J Cardiovasc Pharmacother.

[22] Hill NR, Sandler B, Bergrath E (2020). A systematic review of network meta-analyses and real-world evidence comparing Apixaban and Rivaroxaban in Nonvalvular atrial fibrillation. Clin Appl Thromb Hemost.

[23] Imagawa H, Ryugo M, Shikata F (2009). Coagulant activity during one year after Bioprosthetic aortic valve replacement. Interact Cardiovasc Thorac Surg.

[24] Hasan-Ali H, Mosad E (2015). Changes in platelet, coagulation, and fibrinolytic activities in mitral stenosis after percutaneous mitral Valvotomy: role of hemodynamic changes and systemic inflammation. Clin Appl Thromb Hemost.

[25] Philippart R, Brunet-Bernard A, Clementy N (2016). Oral anticoagulation, stroke and thromboembolism in patients with atrial fibrillation and valve Bioprosthesis. *Thromb Haemost*.

[26] Maggioni AP, Dondi L, Andreotti F (2020). Four-year trends in oral anticoagulant use and declining rates of ischemic stroke among 194,030 atrial fibrillation patients drawn from a sample of 12 million people. Am Heart J.

[27] Harrison SL, Buckley BJR, Ritchie LA (2022). Oral anticoagulants and outcomes in adults ≥80 years with atrial fibrillation: A global Federated health network analysis. J Am Geriatr Soc.

[28] Christersson C, Held C, Modica A (2022). Oral anticoagulant treatment after Bioprosthetic valvular intervention or Valvuloplasty in patients with atrial fibrillation-A SWEDEHEART study. PLoS One.

[29] Jernberg T, Attebring MF, Hambraeus K (2010). The Swedish web-system for Enhancement and development of evidence-based care in heart disease evaluated according to recommended therapies (SWEDEHEART). Heart.

[30] Eikelboom R, Sanjanwala R, Le M-L (2021). Postoperative atrial fibrillation after cardiac surgery: a systematic review and meta-analysis. Ann Thorac Surg.

[31] Thorén E, Wernroth M-L, Christersson C (2020). Compared with matched controls, patients with postoperative atrial fibrillation (POAF) have increased long-term AF after CABG, and POAF is further associated with increased ischemic stroke, heart failure and mortality even after adjustment for AF. Clin Res Cardiol.

[32] Björck F, Sandén P, Renlund H (2015). Warfarin treatment quality is consistently high in both anticoagulation clinics and primary care setting in Sweden. Thromb Res.

[33] Evans M, Carrero J-J, Szummer K (2016). Angiotensin-converting enzyme inhibitors and angiotensin receptor blockers in myocardial infarction patients with renal dysfunction. J Am Coll Cardiol.

