# Supplementary material for: Thromboembolic and bleeding events after valvular intervention in patients with atrial fibrillation
Source: Open Heart. 2024 Jan 29;11(1):e002602. doi: 10.1136/openhrt-2024-002602 (PMC10826562; doi:10.1136/openhrt-2024-002602)
Supplement: online supplemental file 1 [file openhrt-2024-002602supp001.pdf]

Supplementary file

eTable 1. Type of valve intervention, clinical characteristics and comorbidities

**Covariate**	**Source or code(s)**
Type of intervention, Index intervention. Information is collected from NPR and/or SWEDEHEART	
Aortic valve	KVÅ codes FMD00, FMD10, FMD20, FMD30, FMD33, FMD40, FMD96, FMD12, FMD13, FCA50, FCA60, FCA70, FMA10, FMA20, FMA96, FMA00, FMA32, FMC00, FMC10, FMC20, FMC96, FMW96
Mitral valve	KVÅ codes FKD00, FKD10, FKD20, FKD96, FKB00, FKB10, FKB96, FKC00, FKC10, FKC20, FKC30, FKC40, FKC50, FKC60, FKC96, FMW96, FKA00, FKA10, FKA20, FKA32, FKA96, FKW96
Tricuspid valve	KVÅ codes FGE00, FGE10, FGE20, FGE96, FGD00, FGD03, FGD10, FGD30, FGD40, FGD96, FGW96, FGC10, FGC00, FGC96, FGA00, FGA10, FGA32, FGA96
Pulmonary valve	KVÅ codes FJF00, FJF10, FJF12, FJF20, FJF96, FJE00, FJE10, FJE20, FJE30, FJE42, FJE96, FJW96
Biological surgical valve prosthesis	FMD10, FMD20, FMD30, FMD33, FMD40, FKD10, FKD20, FGE10, FGE20, FJF10, FJF20, FCA70
Biological transcatheter valve prosthesis	FMD12, FMD13, FJF12 all in TAVI registry
Biological prosthesis total	FMD10, FMD20, FMD30, FMD33, FMD40, FKD10, FKD20, FGE10, FGE20, FJF10, FJF20, FCA70, FMD12, FMD13, FJF12
Valvuloplasty	FMA20, FMC00, FMC10, FMC20, FMC96, FKB00, FKB10, FKB96, FKC00, FKC10, FKC20, FKC30, FKC40, FKC50, FKC60, FKC96, FGC10, FGC00, FGC96, FJE20, FJE30, FGD00, FGD03, FGD10, FGD30, FGD40, FGD96
CABG	KVÅ codes FNC00, FNC10, FNC20, FNC30, FNC40, FNC50, FNC60, FNC96, FNE00, FNE10, FNE20, FNE96, FNA10, FNA20, FNA96, FND10, FND20, FND96, FNB00, FNB20, FNB96, FNH00, FNH10, FNH20, FNH96, FNW96
Percutaneous coronary intervention	KVÅ codes FNG00, FNG02, FNG05, FNG10, FNG20, FNG22, FNG30, FNG96. Information collected up to 3 months before index intervention
Age at intervention	From the SWEDEHEART registry
Gender	From the SWEDEHEART registry
Smoking	From the SWEDEHEART registry
Diabetes mellitus	ICD codes I E10, E11, E12, E13, E14
Hypertension	ICD codes I10, I11, I12, I13, I15
Heart failure	ICD codes I42, I50, I110, I255, I130, I132, K761
Atrial fibrillation	ICD code I48
COPD	ICD codes J43, J44

Previous ischemic stroke	ICD code I63
Previous systemic embolism	ICD code I74
Previous venous thromboembolism	ICD codes I801, I802, I82, I26, O882, O888
Previous hemorrhagic stroke	ICD code I61
Previous major bleeding	ICD codes; Intracranial bleeding ICD codes: I60, I61, I62, S064, S065, S066. Gastrointestinal bleeding ICD codes: K226, K250, K252, K254, K256, K260, K262, K264, K266, K270, K272, K274, K276, K280, K282, K284, K286, K290, K625, K661, K920, K921, K922, I850, I983. Urogenital bleeding ICD codes N02, R319, N939, N950, N501A. Other bleeding ICD codes H113, H313, H356, H431, H450, H922, I312, J942, M250, R04, R58, T810, D500, D629.
Previous myocardial infarction	ICD codes I21, I22, I252
Previous peripheral vascular disease	ICD codes I70, I71, I72, I73
Previous CKD	ICD codes N18, N17, N19, Z992
Medical treatment	According to appendix of ATC codes
STS score, EURO score	Information is collected from SWEDEHEART registry

eTable 2. CHA2DS2-VASc score

Points	Condition	Definition
1	Heart Failure	I50, I110, I130, I132, I255, K761, I42-43
1	Hypertension	I10-15<br><br>ATC codes (ONLY if NOT co-occur with HEART FAILURE) Treatment with at least two of the following classes (I-VI) of antihypertensive drugs:<br>• I· Alpha adrenergic blockers (C02A, C02B, C02C)<br>• II· Non-loop diuretics (C02DA, C02L, C03A, C03B, C03D, C03E, C03X, C07C, C07D, C08G, C09BA, C09DA, C09XA52)<br>• III· Vasodilators (C02DB, C02DD, C02DG, C04, C05) IV· Beta blockers (C07) V· Calcium channel blockers (C08)<br>• VI· Renin-angiotensin system inhibitors (C09A) Or at least one antihypertensive combination drug (C07B, C07C, C07D, C07F, C08G, C09B, C09DA, C09DB, C09DX01, C09DX04, C09XA52)<br>*codes may appear several times
1	Diabetes mellitus	E10-14 or use of antidiabetic drug (ATC codes beginning with A10)
2	Stroke, TIA or systemic embolism	Hospitalization with I61, I63, I64 or I74 as principal or first secondary diagnosis, and any G45 diagnosis
1	Vascular Disease (myocardial infarction or peripheral arterial disease)	Hospitalization with I21 or I22 as principal or first secondary diagnosis, I70-73
1	Female	
1	Age 65-<75 years	
2	Age≥ 75 years	

eTable 3. HAS-BLED

**Points**	**Condition**	**Definition**
**1**	Hypertension	Use definition for "Hypertension" from eTable 1.
**1**	Abnormal kidney function	N18-19, procedure codes DR016, DR024, KAS00, KAS10, KAS20
**1**	Abnormal liver function:	K70-77, procedure codes JJB, JJC
**1**	Stroke or TIA	Hospitalization with I61, I63, or I64 as principal or first secondary diagnosis, and any G45 diagnosis
**1**	Any bleeding other than haemorrhagic stroke	Any of intracranial, gastrointestinal, urogenital or other bleeding (see eTable 1), excluding codes for haemorrhagic stroke I60, I61, I690-I692
	Labile INR	
**1**	Age≥ 65 years	1 point for age 65 years or older
**1**	Alcohol/ Drug Not available Therapy	E244, F10, G312, G621, G721, I426, K292, K70, K860, O354, P043, Q860, T51, Y90-91, Z502, Z714

eTable 4. Oral anticoagulant treatment

**Drug type**	**Drug name**	**ATC code**
Vitamin K antagonist	Warfarin	B01AA03
NOAC	Apixaban	B01AF02
	Dabigatran	B01AE07
	Edoxaban	B01AF03
	Rivaroxaban	B01AF01

eTable 5. Outcome events

**Outcome**	**ICD code(s)**
**The composite cardiovascular outcome event**; cardiovascular death, ischemic stroke and systemic embolization. (Primary analysis)	See respective ICD codes below
Ischemic stroke	I63
Systemic embolization	I74
**Major bleeding event**; including intracranial bleeding, gastrointestinal bleeding, urogenital bleeding and other bleeding (see below)	See respective ICD codes below
Intracranial bleeding	I60, I61, I62, S064, S065, S066
Gastrointestinal bleeding	K226, K250, K252, K254, K256, K260, K262, K264, K266, K270, K272, K274, K276, K280, K282, K284, K286, K290, K625, K661, K920, K921, K922, I850, I983
Urogenital bleeding	N02, R319, N939, N950, N501A
Other bleeding	H113, H313, H356, H431, H450, H922, I312, J942, M250, R04, R58, T810, D500, D629.

eTable 6. Baseline characteristics group B.

	All Patients	Warfarin	NOAC	No OAC
	N=5560	N=3292	N=642	N=1626
Age – median (IQR)	74.0 (67-80)	74.0 (67-79)	75.0 (68-81)	74.0 (67-81)
Female sex – median (IQR)	1977 (35.6)	1114 (33.8)	241 (37.5)	622 (38.3)
Creatinine (µmol/L) – median (IQR)	99.0 (80-130)	98 (79-128)	95 (79-120)	102 (81-139)
**Medical history**				
Heart failure – no. (%)	1826 (32.8)	1097 (33.3)	206 (32.1)	523 (32.2)
Hypertension – no. (%)	2800 (50.4)	1654 (50.2)	324 (50.5)	822 (50.6)
Diabetes mellitus – no. (%)	878 (15.8)	510 (15.5)	98 (15.3)	270 (16.6)
Ischemic stroke – no. (%)	344 (6.2)	196 (6.0)	38 (5.9)	110 (6.8)
TIA – no. (%)	223 (4.0)	136 (4.1)	24 (3.7)	63 (3.9)
Myocardial infarction – no. (%)	813 (14.6)	456 (13.9)	91 (14.2)	266 (16.4)
Peripheral artery disease – no. (%)	398 (7.2)	214 (6.5)	47 (7.3)	137 (8.4)
Systemic embolism – no. (%)	32 (0.6)	25 (0.8)	1 (0.2)	6 (0.4)
Chronic kidney disease – no. (%)	331 (6.0)	164 (5.0)	29 (4.5)	138 (8.5)
Cancer – no. (%)	262 (4.7)	141 (4.3)	31 (4.8)	90 (5.5)
Mitral stenosis – no. (%)	88 (1.6)	63 (1.9)	2 (0.3)	23 (1.4)
Intracranial bleeding – no. (%)	67 (1.2)	31 (0.9)	8 (1.2)	28 (1.7)
Gastrointestinal bleeding – no. (%)	324 (5.8)	153 (4.6)	48 (7.5)	123 (7.6)
Other major bleeding – no. (%)	353 (6.3)	182 (5.5)	32 (5.0)	139 (8.5)
CHA_2DS_2-VASc score, mean	3.1	3.1	3.1	3.2
HAS-BLED, mean	1.6	1.5	1.6	1.6
**Valve intervention type**				
Surgical biological valve prosthesis – no. (%)	3159 (56.8)	1856 (56.4)	358 (55.8)	945 (58.1)
Surgical valvuloplasty – no. (%)	1363 (24.5)	925 (28.1)	118 (18.4)	320 (19.7)
TAVI – no. (%)	1038 (18.7)	511 (15.5)	166 (25.9)	361 (22.2)
**Valve position**				
Aortic position – no. (%)	3984 (71.7)	2203 (66.9)	525 (81.8)	1256 (77.2)
Mitral position – no. (%)	1622 (29.2)	1135 (34.5)	131 (20.4)	356 (21.9)
Tricuspid position – no. (%)	616 (11.1)	406 (12.3)	41 (6.4)	16 (10.4)
Pulmonary position – no. (%)	25 (0.4)	11(0.3)	0 (0.0)	14 (0.9)

eTable 7 Oral anticoagulant exposure and outcome during follow up in the surgical biological valve prosthesis subgroup

Time-period	Discharge to 3 months post intervention			3 to 12 months post intervention		
	**Warfarin**	**NOAC**	**No OAC**	**Warfarin**	**NOAC**	**No OAC**
***Composite CV$^{\alpha}$-event**	46 (380) 12.1	10 (56) 17.8	51 (182) 28.0	79(1247) 6.3	17 (306) 5.6	45 (924) 4.9
***CV$^{\alpha}$-death**	23 (385) 6.0	8 (57) 14.1	38 (183) 20.7	41 (1262) 3.2	6 (313) 1.9	28 (937) 3.0
***Ischemic stroke/ SEE$^{\beta}$**	23 (380) 6.1	3 (56) 5.3	13 (182) 7.1	38 (1246) 3.0	11 (306) 3.6	21 (924) 2.3
***Major bleeding event**	43 (377) 11.4	8 (56) 14.3	28 (180) 15.6	80 (1645) 4.9	14 (437) 3.2	47 (1189) 4.0

*Expressed as frequencies (patient years) incidence rate, %/year. $^{\alpha}$CV; cardiovascular, $^{\beta}$SE; systemic embolism

eTable 8 Interaction effect of valve position of surgical biological prosthesis (aortic or mitral) and OAC treatment on risk of occurrence of outcome events.

	Biological prosthesis in aortic position and OAC		Biological prosthesis in mitral position and OAC	
	0 - 3 months	3 – 12 months	0 - 3 months	3 – 12 months
CV death, stroke, systemic embolism – p-value crude model (p-value adjusted model)	0.75 (0.79)	0.57 (0.86)	0.47 (0.81)	0.23 (0.20)
Stroke, systemic embolism – p-value crude model (p-value adjusted model)	0.88 (0.87)	0.83 (0.97)	0.93 (0.93)	0.57 (0.56)
CV death – p-value crude model (p-value adjusted model)	0.99 (0.97)	0.66 (0.80)	0.67 (0.89)	0.75 (0.66)
Major bleeding – p-value crude model (p-value adjusted model)	0.65 (0.63)	0.78 (0.67)	0.70 (0.56)	0.39 (0.25)

eTable 9 Oral anticoagulant exposure and outcome during follow up in the TAVI subgroup

Time-period	**Discharge to 3 months post intervention**			**3 to 12 months post intervention**		
	**Warfarin**	**NOAC**	**No OAC**	**Warfarin**	**NOAC**	**No OAC**
***Composite CV$^{\alpha}$-event**	27 (138.5) 19.5	3 (38.7) 7.7	23 (77.9) 29.5	40 (405.5) 9.9	17 (131.6) 12.9	38 (276.7) 13.7
***CV$^{\alpha}$-death**	20 (139.7) 14.3	3 (38.9) 7.7	16 (78.8) 20.3	34 (409.3) 8.3	15 (132.8) 11.3	31 (281.0) 11.0
***Ischemic stroke/ SEE$^{\beta}$**	9 (138.5) 6.5	0 (38.7) 0.0	7 (77.9) 9.0	8 (405.5) 2.0	2 (131.6) 1.5	10 (276.7) 3.6
***Major bleeding event**	10 (138.5) 7.2	2 (38.7) 5.2	14 (76.8) 18.2	13 (405.1) 3.2	7 (129.3) 5.4	13 (274.4) 4.7

*Expressed as frequencies (patient years) incidence rate, %/year. $^{\alpha}$CV; cardiovascular, $^{\beta}$SE; systemic embolism

eFigure 1

	PY	Events (%/year)	HR−unadj (CI 95%)	HR−adj (CI 95%)
**CV−death/stroke/SSE**				
OAC	835.0	107 (12.8)		
No OAC	314.0	84 (26.8)	1.88 (1.41-−2.50)	1.71 (1.27-−2.29)
**Stroke/SE**				
OAC	835.0	47 (5.6)		
No OAC	314.0	22 (7.0)	1.11 (0.67-−1.84)	1.11 (0.66-−1.86)
**CV−death**				
OAC	844.3	63 (7.5)		
No OAC	316.7	62 (19.6)	2.37 (1.67-−3.37)	2.06 (1.44-−2.96)
**Bleeding**				
OAC	830.8	82 (9.9)		
No OAC	309.3	52 (16.8)	1.52 (1.07-−2.15)	1.35 (0.94-−1.92)

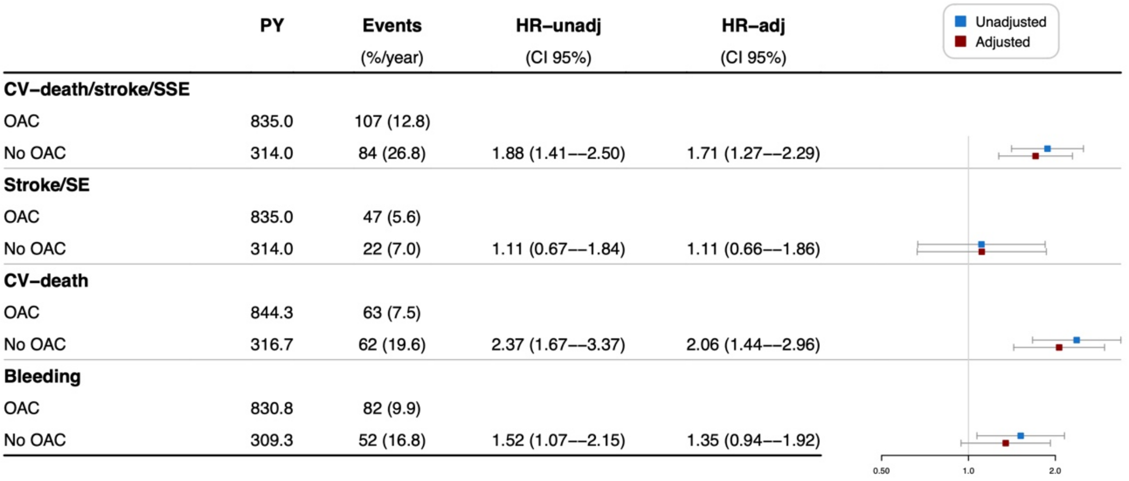

The event rates of the composite CV outcome (CV death, ischemic stroke or systemic embolism); ischemic stroke or systemic embolism; CV death, and major bleeding, and hazard ratios (HR) (95% CI) for no OAC versus OAC during the first 3 months after discharge from valvular intervention. The adjusted model included baseline characteristics and comorbidities (age, sex, eGFR, hypertension, diabetes mellitus, heart failure, myocardial infarction, ischemic stroke, TIA, unspecified stroke, peripheral arterial disease, systemic embolism, venous thromboembolism and major bleeding event).

Abbreviations: PY; patient years; %/year; percent per person year; HR-unadj;, Hazard ratio unadjusted; HR-adj; Hazard ratio adjusted; CI; confidence interval; CV death; cardiovascular death; SE; systemic embolism; NOAC; non-vitamin K oral anticoagulant; OAC; oral anticoagulant.

eFigure 2

	PY	Events (%/year)	HR–unadj (CI 95%)	HR–adj (CI 95%)
**CV–death/stroke/SSE**				
OAC	2835.8	185 (6.5)		
No OAC	1548.5	100 (6.5)	1.12 (0.88-–1.43)	1.12 (0.88-–1.44)
**Stroke/SE**				
OAC	2835.8	78 (2.8)		
No OAC	1548.5	39 (2.5)	1.03 (0.70-–1.52)	1.07 (0.72-–1.59)
**CV–death**				
OAC	2873.6	109 (3.8)		
No OAC	1568.9	69 (4.4)	1.31 (0.97-–1.78)	1.23 (0.91-–1.68)
**Bleeding**				
OAC	2831.9	116 (4.1)		
No OAC	1534.6	51 (3.3)	0.92 (0.66-–1.28)	0.89 (0.64-–1.24)

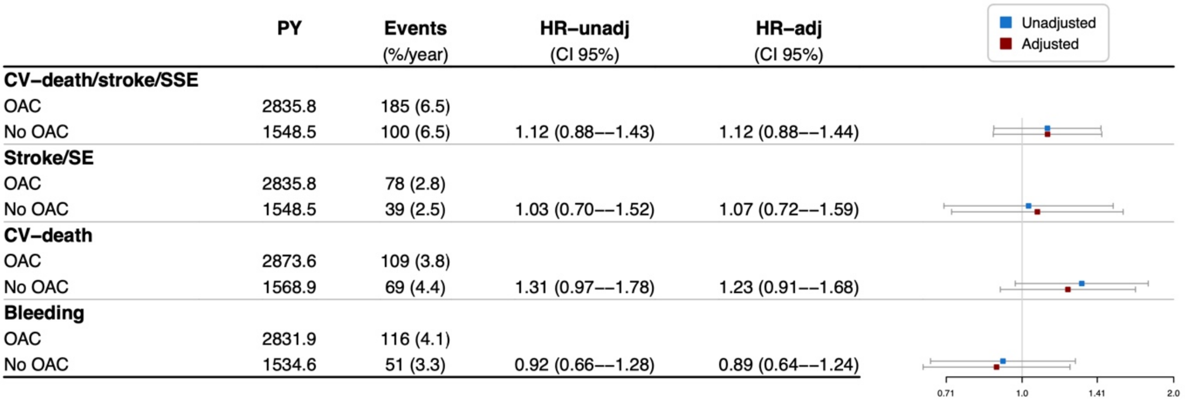

The event rates of the composite CV outcome (CV death, ischemic stroke or systemic embolism); ischemic stroke or systemic embolism; CV death, and major bleeding, and hazard ratios (HR) (95% CI) no OAC versus OAC from 3 to 12 months after discharge from valvular intervention. The adjusted model included baseline characteristics (age, sex, eGFR, hypertension, diabetes mellitus, heart failure, myocardial infarction, ischemic stroke, TIA, unspecified stroke, peripheral arterial disease, systemic embolism, venous thromboembolism and major bleeding event).

Abbreviations: PY; patient years; %/year; percent per person year; HR-unadj; Hazard ratio unadjusted; HR-adj; Hazard ratio adjusted; CI; confidence interval; CV death; cardiovascular death; SE; systemic embolism; NOAC; non-vitamin K oral anticoagulant; OAC; oral anticoagulant.

eFigure 3

	PY	Events (%/year)	HR–unadj (CI 95%)	HR–adj (CI 95%)
**CV–death/stroke/SSE**				
Warfarin	379.9	46 (12.1)		
NOAC	56.2	10 (17.8)	1.49 (0.75 – 2.95)	1.60 (0.80 – 3.17)
No OAC	181.9	51 (28.0)	1.97 (1.32 – 2.94)	1.88 (1.25 – 2.84)
**Stroke/SE**				
Warfarin	379.9	23 (6.1)		
NOAC	56.2	3 (5.3)	0.90 (0.27 – 2.98)	0.89 (0.27 – 2.97)
No OAC	181.9	13 (7.1)	0.96 (0.48 – 1.91)	0.95 (0.47 – 1.91)
**CV–death**				
Warfarin	385.1	23 (6.0)		
NOAC	56.9	8 (14.1)	2.38 (1.07 – 5.32)	2.58 (1.15 – 5.78)
No OAC	183.5	38 (20.7)	3.01 (1.79 – 5.06)	2.82 (1.65 – 4.82)
**Bleeding**				
Warfarin	377.3	43 (11.4)		
NOAC	55.8	8 (14.3)	1.25 (0.59 – 2.67)	1.25 (0.58 – 2.66)
No OAC	179.6	28 (15.6)	1.16 (0.72 – 1.88)	1.13 (0.68 – 1.87)

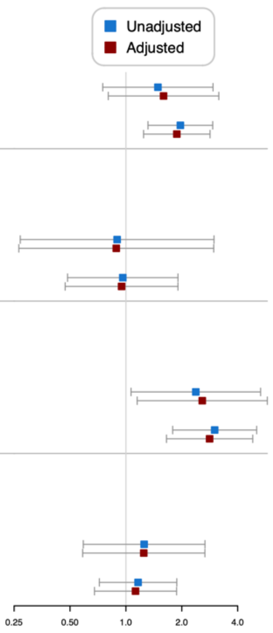

The event rates of the composite CV outcome (CV death, ischemic stroke or systemic embolism); ischemic stroke or systemic embolism; CV death, and major bleeding, and hazard ratios (HR) (95% CI) for NOAC versus warfarin and no OAC versus warfarin during the first 3 months after discharge from surgical valve replacement with biological prosthesis. The adjusted model included baseline characteristics and comorbidities (age, sex, eGFR, hypertension, diabetes mellitus, heart failure, venous thromboembolism, myocardial infarction, unspecified stroke, peripheral arterial disease, previous major bleeding, ischemic stroke, TIA, systemic embolism and for the outcome major bleeding also ongoing antiplatelet treatment).

Abbreviations: PY; patient years; %/year; percent per person year; HR-unadj;, Hazard ratio unadjusted; HR-adj; Hazard ratio adjusted; CI; confidence interval; CV death; cardiovascular death; SE; systemic embolism; NOAC; non-vitamin K oral anticoagulant; OAC; oral anticoagulant.

eFigure 4

	PY	Events (%/year)	HR–unadj (CI 95%)	HR–adj (CI 95%)
**CV–death/stroke/SSE**				
Warfarin	1246.5	79 (6.3)		
NOAC	306.3	17 (5.6)	0.90 (0.53 – 1.52)	1.06 (0.63 – 1.81)
No OAC	923.9	45 (4.9)	0.89 (0.61 – 1.28)	0.95 (0.65 – 1.39)
**Stroke/SE**				
Warfarin	1246.5	38 (3.0)		
NOAC	306.3	11 (3.6)	1.18 (0.60 – 2.30)	1.41 (0.72 – 2.78)
No OAC	923.9	21 (2.3)	0.82 (0.48 – 1.41)	0.93 (0.54 – 1.61)
**CV–death**				
Warfarin	1262.1	41 (3.2)		
NOAC	312.5	6 (1.9)	0.62 (0.27 – 1.47)	0.73 (0.31 – 1.72)
No OAC	936.5	28 (3.0)	1.12 (0.69 – 1.81)	1.04 (0.63 – 1.72)
**Bleeding**				
Warfarin	1644.8	80 (4.9)		
NOAC	437.0	14 (3.2)	0.65 (0.37 – 1.15)	0.49 (0.23 – 1.08)
No OAC	1189.8	47 (4.0)	0.89 (0.62 – 1.28)	0.73 (0.46 – 1.16)

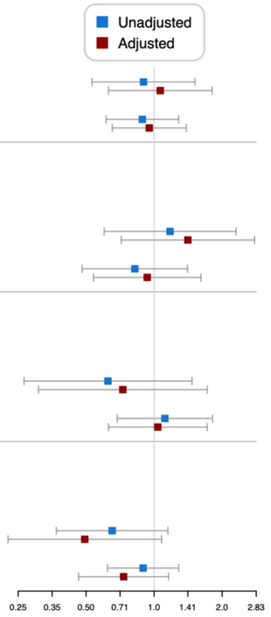

The event rates of the composite CV outcome (CV death, ischemic stroke or systemic embolism); ischemic stroke or systemic embolism; CV death, and major bleeding, and hazard ratios (HR) (95% CI) for NOAC versus warfarin and no OAC versus warfarin from 3 to 12 months after discharge from surgical valve replacement with biological prosthesis. The adjusted model included baseline characteristics (age, sex, eGFR, hypertension, diabetes mellitus, heart failure, myocardial infarction, ischemic stroke, TIA, unspecified stroke, peripheral arterial disease, previous major bleeding event, systemic embolism, venous thromboembolism and for the outcome major bleeding also ongoing antiplatelet treatment). Abbreviations: PY; patient years; %/year; percent per person year; HR-unadj; Hazard ratio unadjusted; HR-adj; Hazard ratio adjusted; CI; confidence interval; CV death; cardiovascular death; SE; systemic embolism; NOAC; non-vitamin K oral anticoagulant; OAC; oral anticoagulant.

eFigure 5

	PY	Events (%/year)	HR–unadj (CI 95%)	HR–adj (CI 95%)
**CV–death/stroke/SSE**				
Warfarin	138.5	27 (19.5)		
NOAC	38.7	3 (7.7)	0.39 (0.12−−1.30)	0.40 (0.12−−1.31)
No OAC	77.9	23 (29.5)	1.44 (0.82−−2.51)	1.23 (0.70−−2.18)
**Stroke/SE**				
Warfarin	138.5	9 (6.5)		
NOAC	38.7	0 (0.0)	0.00 ()	0.00 ()
No OAC	77.9	7 (9.0)	1.32 (0.49 – 3.54)	1.00 (0.36 – 2.76)
**CV–death**				
Warfarin	139.7	20 (14.3)		
NOAC	38.9	3 (7.7)	0.53 (0.16 – 1.80)	0.58 (0.17 – 1.96)
No OAC	78.8	16 (20.3)	1.35 (0.70 – 2.60)	1.23 (0.63 – 2.43)
**Bleeding**				
Warfarin	138.5	10 (7.2)		
NOAC	38.7	2 (5.2)	0.71 (0.16 – 3.24)	0.79 (0.17 – 3.69)
No OAC	76.8	14 (18.2)	2.36 (1.05 – 5.32)	2.39 (1.01 – 5.68)

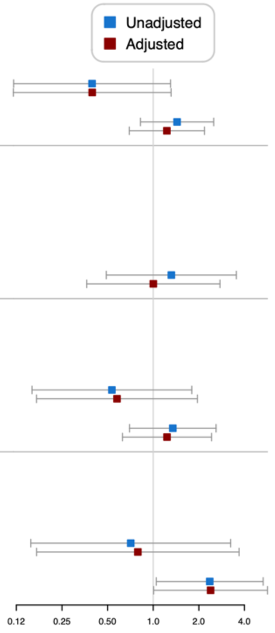

The event rates of the composite CV outcome (CV death, ischemic stroke or systemic embolism); ischemic stroke or systemic embolism; CV death, and major bleeding, and hazard ratios (HR) (95% CI) for NOAC versus warfarin and no OAC versus warfarin during the first 3 months after discharge after TAVI. The adjusted model included baseline characteristics and comorbidities (age, sex, eGFR, hypertension, diabetes mellitus, heart failure, venous thromboembolism, myocardial infarction, unspecified stroke, peripheral arterial disease, previous major bleeding, ischemic stroke, TIA, systemic embolism and for the outcome major bleeding also ongoing antiplatelet treatment).

Abbreviations: PY; patient years; %/year; percent per person year; HR-unadj;, Hazard ratio unadjusted; HR-adj; Hazard ratio adjusted; CI; confidence interval; CV death; cardiovascular death; SE; systemic embolism; NOAC; non-vitamin K oral anticoagulant; OAC; oral anticoagulant, TAVI; transcatheter aortic valve implantation.

eFigure 6

	PY	Events (%/year)	HR–unadj (CI 95%)	HR–adj (CI 95%)
**CV–death/stroke/SSE**				
Warfarin	405.5	40 (9.9)		
NOAC	131.6	17 (12.9)	1.23 (0.70––2.16)	1.26 (0.71––2.23)
No OAC	276.7	38 (13.7)	1.47 (0.94––2.29)	1.34 (0.86––2.11)
**Stroke/SE**				
Warfarin	405.5	8 (2.0)		
NOAC	131.6	2 (1.5)	0.72 (0.15 – 3.41)	0.87 (0.18 – 4.12)
No OAC	276.7	10 (3.6)	1.94 (0.76 – 4.91)	1.92 (0.75 – 4.92)
**CV–death**				
Warfarin	409.3	34 (8.3)		
NOAC	132.8	15 (11.3)	1.27 (0.69 – 2.33)	1.28 (0.69 – 2.35)
No OAC	281.0	31 (11.0)	1.39 (0.86 – 2.27)	1.24 (0.76 – 2.03)
**Bleeding**				
Warfarin	405.1	13 (3.2)		
NOAC	129.3	7 (5.4)	1.59 (0.63 – 3.98)	1.74 (0.69 – 4.42)
No OAC	274.4	13 (4.7)	1.55 (0.72 – 3.35)	1.63 (0.71 – 3.74)

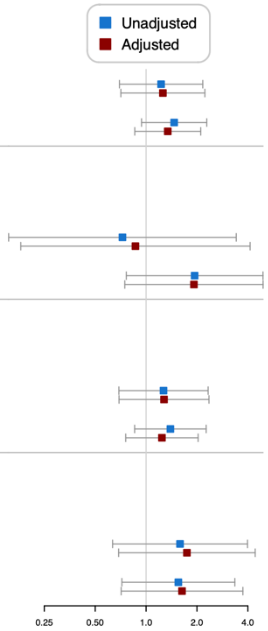

The event rates of the composite CV outcome (CV death, ischemic stroke or systemic embolism); ischemic stroke or systemic embolism; CV death, and major bleeding, and hazard ratios (HR) (95% CI) for NOAC versus warfarin and no OAC versus warfarin from 3 to 12 months after discharge after TAVI. The adjusted model included baseline characteristics and comorbidities (age, sex, eGFR, hypertension, diabetes mellitus, heart failure, venous thromboembolism, myocardial infarction, unspecified stroke, peripheral arterial disease, previous major bleeding, ischemic stroke, TIA, systemic embolism and for the outcome major bleeding also ongoing antiplatelet treatment).

Abbreviations: PY; patient years; %/year; percent per person year; HR-unadj;, Hazard ratio unadjusted; HR-adj; Hazard ratio adjusted; CI; confidence interval; CV death; cardiovascular death; SE; systemic embolism; NOAC; non-vitamin K oral anticoagulant; OAC; oral anticoagulant, TAVI; transcatheter aortic valve implantation.
